# Phosphorus-Modified Palladium and Tungsten Carbide/Mesoporous Carbon Composite for Hydrogen Oxidation Reaction of Proton Exchange Membrane Fuel Cells

**DOI:** 10.3390/nano14121024

**Published:** 2024-06-13

**Authors:** Ganghong Bae, Woo Jin Byun, Jin Ho Lee, Min Hee Lee, Yeji Choi, Jae Young Kim, Duck Hyun Youn

**Affiliations:** 1 Department of Chemical Engineering, Pohang University of Science & Technology (POSTECH), Pohang 37673, Republic of Korea; bgh711@postech.ac.kr; 2 School of Energy and Chemical Engineering, Ulsan National Institute of Science and Technology (UNIST), Ulsan 44919, Republic of Korea; wjenergy@unist.ac.kr (W.J.B.); miraculum@unist.ac.kr (J.H.L.); photon@unist.ac.kr (M.H.L.); 3 Department of Chemical Engineering, Department of Integrative Engineering for Hydrogen Safety, Kangwon National University, Chuncheon 24341, Republic of Korea; yeahd27@kangwon.ac.kr; 4 Korea Research Institute of Chemical Technology, Daejeon 34114, Republic of Korea

**Keywords:** hydrogen oxidation reaction, mesoporous carbon, tungsten carbide/mesoporous carbon composite, phosphorus-modified palladium

## Abstract

A composite material of tungsten carbide and mesoporous carbon was synthesized by the sol-gel polycondensation of resorcinol and formaldehyde, using cetyltrimethylammonium bromide as a surfactant and Ludox HS-40 as a porogen, and served as a support for Pd-based electrodes. Phosphorus-modified Pd particles were deposited onto the support using an NH_3_-mediated polyol reduction method facilitated by sodium hypophosphite. Remarkably small Pd nanoparticles with a diameter of ca. 4 nm were formed by the phosphorus modification. Owing to the high dispersion of Pd and its strong interaction with tungsten carbide, the Pd nanoparticles embedded in the tungsten carbide/mesoporous carbon composite exhibited a hydrogen oxidation activity approximately twice as high as that of the commercial Pt/C catalyst under the anode reaction conditions of proton exchange membrane fuel cells.

## 1. Introduction

Transition metal carbides (TMCs) have garnered extensive interest due to their remarkable physicochemical properties, including hardness, wear resistance, superconductivity, and high chemical stability [1]. Notably, TMCs exhibit platinum-like behavior in catalytic reactions such as hydrogenation and hydrodesulfurization, attributed to their electronic structures akin to noble metals [2,3]. Recently, TMCs have been employed in various energy applications, including electrochemical water splitting [4,5], fuel cells [6,7], solar cells [8,9], lithium-ion batteries [10,11], and photoelectrochemical cells [12,13]. Specifically, TMCs have been explored as potential candidates for anode and cathode electrocatalysts in low-temperature fuel cells, such as proton exchange membrane fuel cells (PEMFCs) and direct methanol fuel cells (DMFCs). Despite these advances, their catalytic activities remain inferior to those of commercial Pt/C catalysts. Recent studies have demonstrated that tungsten carbides (WCs) function as effective supports in promoting the electrochemical activities of other metals [6,14]. For instance, the Pt/WC catalysts have shown higher methanol oxidation activity, improved tolerance to CO poisoning, and enhanced oxygen reduction activity compared to the conventional Pt/C catalysts [6,15,16,17], indicating a synergistic effect between WC and Pt. To further enhance the catalytic activity, WCs with various shapes and structures have been synthesized by different methods and applied in various catalytic reactions [18,19,20]. However, the inherently low surface area of WC limits its effectiveness as a support. This limitation has been addressed by using ordered mesoporous carbon (m-C) and WC composites, which significantly improve performance in some reactions due to their higher surface area, larger pore volumes, and narrower port size distribution [21,22]

Palladium (Pd) has been investigated as an alternative catalyst to Pt in certain fuel cell reactions, including formic acid and ethanol oxidation reactions [23,24,25,26]. Pd shares similar valence shell electronic configurations and lattice constants with Pt but is less expensive and more abundant [27]. Moreover, the Pd catalysts are highly methanol-tolerant, making them promising alternatives for the oxygen reduction reaction [28,29]. However, their widespread adoption has been hampered by serious dissolution in the acidic conditions typical of PEMFCs (pH < 2) [30]. In addition, the conventional polyol method often fails to yield well-dispersed Pd catalysts. There have been several attempts to produce small and well-dispersed Pd nanoparticles using protective stabilizers [31,32,33]. Unfortunately, these stabilizers tend to adsorb onto the surface of the nanoparticles, diminishing the catalytic activity of Pd. Recently, phosphorus modification was reported to be an effective method to synthesize small, well-dispersed Pd particles that showed high catalytic activity in formic acid/ethanol oxidation reactions and the oxygen reduction reaction [27,34,35].

In our previous studies, we deposited Pd on WC and confirmed a synergistic effect between them as an electrocatalyst for the hydrogen oxidation reaction (HOR) [36]. In addition to enhanced activity, the stability of Pd is also improved, exhibiting minimal dissolution under acidic PEMFC conditions. Nevertheless, the HOR activity of the Pd/WC catalyst was significantly lower than that of the commercial Pt/C (E-TEK) catalyst, primarily due to the formation of large Pd and WC particles.

**Scheme 1:**
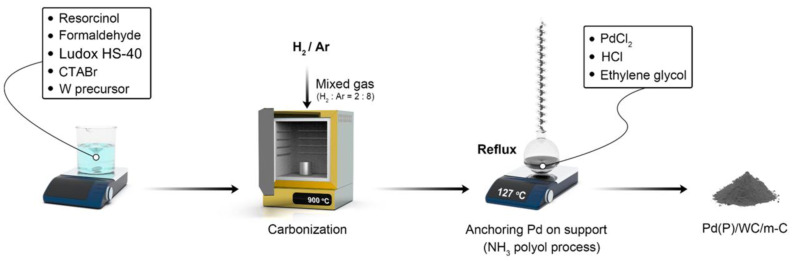
Schematic diagram summarizing the synthesis steps of the Pd(P)/WC/m-C catalysts.

To address these issues, this study focuses on the synthesis of a tungsten carbide/mesoporous carbon composite (WC/m-C) as a support and the subsequent loading of phosphorus-modified Pd nanoparticles using an NH_3_-mediated polyol method in the presence of sodium hypophosphite (Scheme 1). The choice of phosphorus-modified palladium (Pd(P)) and tungsten carbide/mesoporous carbon (WC/m-C) composites is based on their potential to improve the catalytic activity and stability of Pd in PEMFC applications.

The notable aspect of this work lies in the successful synthesis of a high surface area support of WC/m-C composite and P-modified Pd particles (Pd(P)) with an approximate size of 4 nm. The resulting Pd(P)/WC/m-C catalyst exhibited a two-fold increase in HOR activity compared to the commercial Pt/C (E-TEK) catalyst. This result highlights the potential of non-Pt catalysts to achieve high performance in HOR applications.

This study aims to elucidate the effects of mesoporous carbon and phosphorus modification on the physical properties of the electrocatalyst and its performance in HOR under PEMFC conditions. The anticipated outcome of this approach is to provide a more cost-effective and stable alternative to Pt-based catalysts, addressing both the economic and durability challenges associated with current fuel cell technologies.

## 2. Materials and Methods

### 2.1. Synthesis of m-C

The m-C was synthesized via sol-gel polycondensation of resorcinol and formaldehyde (37 wt% in an aqueous solution stabilized by 10–15% methanol) with the addition of a cetyltrimethylammonium bromide (CTABr) surfactant and Ludox HS-40. Resorcinol (0.6 g) and formaldehyde (0.9 mL) were mixed with distilled water. 1M NaOH (0.545 mL), CTABr (198.6 mg), and Ludox HS-40 (2.31 mL) were added to the solution, which was subsequently refluxed at 358 K for 24 h. Following the polymerization reaction, the mixture was filtered, washed with water, and dried in an oven at 373 K overnight. The resultant mixture was then carbonized at 1173 K for 3 h under Ar flow (100 sccm). After cooling, the mesoporous carbon was obtained by dissolving the Ludox template in an HF solution.

### 2.2. Synthesis of WC/m-C

The WC/m-C was prepared using a method similar to that for m-C. Ammonium metatungstate (AMT, 1.343 g) as a source for WC was added to the initial solution containing resorcinol and formaldehyde. After the polymerization reaction with NaOH, CTABr, and Ludox HS-40, the mixture was carbonized at 1173 K for 3 h under a mixed gas flow of H_2_ (20 sccm) and Ar (80 sccm). After removing the silica template (Ludox HS-40, Sigma Aldrich, St. Louis, MO, USA) with HF solution, the WC/m-C composite was obtained.

### 2.3. Pd or Pd(P) Nanoparticle Loading onto Support Materials

Carbon black (Vulcan XC-72R, Cabot, Alpharetta, GA, USA), m-C, and WC/m-C were employed as support materials for Pd nanoparticles, with a loading of 20 wt% Pd using an NH_3_-mediated polyol procedure [24]. In a typical synthesis, PdCl_2_ (5 wt% solution in 10 wt% HCl, 628.75 µL) was added to 25 mL of ethylene glycol under vigorous stirring. After adding 0.5 mL of ammonia solution, the solution’s pH was adjusted to 10 with 1M NaOH, followed by the addition of 80 mg of the support materials. The mixture was then refluxed at 400 K for 3 h. Upon cooling, the mixtures were filtered, washed with water and ethanol, and dried at 373 K.

P-modified Pd nanoparticles (Pd(P)) were deposited onto the support materials using the previously described Pd loading procedure, except that a sodium hypophosphite (NaH_2_PO_2_) solution with a molar ratio of Pd:P = 1:60 was added to the ethylene glycol solution containing PdCl_2_, ammonia, and NaOH.

### 2.4. Catalyst Characterization

The crystal structures of the prepared catalysts were analyzed by X-ray diffraction (XRD, PANalytical, Malvern, UK, PW 3040/60 X’pert). The structural morphologies were examined using cs-corrected high-resolution scanning transmission electron microscopy (HR-TEM, JEOL, Tokyo, Japan, JEM-2100F) equipped with energy-dispersive spectroscopy (EDS). Nitrogen adsorption–desorption isotherms were measured at 77 K using Nano Porosity-XQ (Mirae Scientific Instruments, Gwangju, Republic of Korea). The N_2_ isotherm-derived specific surface area and pore size distribution (PSD) were determined by the Brunauer–Emmett–Teller (BET) and Barrett–Joyner–Halenda (BJH) methods, respectively. X-ray photoelectron spectroscopy (XPS) was used to analyze the functional groups containing P. The compositions of the catalysts were investigated using inductively coupled plasma (ICP, Agilent, Santa Clara, US, 5900) and elemental analysis (Elementar, Langenselbold, Germany, Vario MICRO).

### 2.5. Electrochemical Tests

Hydrogen oxidation reaction tests were conducted in a conventional three-electrode cell containing an N_2_-saturated 1M H_2_SO_4_ solution using a potentiostat (Ivium Technologies, Eindhoven, The Netherlands). A Pt wire and an Ag/AgCl electrode served as the counter and reference electrodes, respectively. All potentials were reported relative to the reversible hydrogen electrode (RHE). The working electrodes were prepared by mixing 20 mg of the catalyst in 1 mL of distilled water and 10 μL of 5% Nafion solution, then pipetting out 5 μL of the catalyst slurry onto a glassy carbon electrode (0.0707 cm^2^). An additional 3 μL of Nafion solution was dropped on top to secure the catalyst. Cyclic voltammetry (CV) tests were performed from 0 V to 1.2 V at a scan rate of 50 mV/s. To determine the electrochemical surface area (ECSA), CO stripping measurements were conducted in Ar-purged electrolyte [36]. A potential of 0.1 V was applied for 5 min to facilitate CO adsorption on the monolayer of the Pd surface. The electrode system was then purged with inert Ar gas to remove any remaining CO, except for the adsorbed CO. The adsorbed CO was subsequently then stripped by scanning between 0.05 and 1.25 V (vs. RHE) at 50 mV/s. The ECSA_CO_ was determined by the following Equation (1):

ECSA_CO_ = Q_CO_/(M_Pd_ × c) × v (1)

where Q_CO_ (μC) is the charge for the CO adsorption, M_Pd_ is the loading of Pd on the GC electrode (mg), v is the scan rate (V/s), and c is the charge required to form a full monolayer of the CO per unit area (420 μC cm^−2^) [37].

## 3. Results

### 3.1. Physical and Electrochemical Properties of m-C and WC/m-C Supports

Before treating Pd-based catalysts, the structural characterizations of the support materials were investigated. Figure S1 shows the XRD patterns for the synthesized m-C and WC/m-C supports. The diffraction peaks of WC/m-C correspond to reference WC patterns (JCPDS PDF #00-051-0939) without any impurity peaks, including tungsten oxides and tungsten metal, confirming the successful fabrication of WC by polycondensation reaction. Conversely, the m-C support shows only broad peaks, suggesting the formation of amorphous carbon.

Figure S2 presents TEM images of the prepared supports. In the m-C support, numerous well-defined mesopores with spherical morphology were observed (Figure S2a). The pore size was estimated to be ca. 15 nm, and graphitic layers were not detected (Figure S2b), which is consistent with the XRD results. The pores observed in the m-C support are induced by the removal of Ludox HS-40 (colloidal silica with an average size of 12 nm). Figure S2c,d show mesopores similar in size to those in m-C, interspersed with various sizes of WC particles in the WC/m-C support. Small WC particles, ca. 10 nm in size, are embedded in the m-C matrix, while larger WC particles are adhered to the m-C.

The textural properties of m-C and WC/m-C were investigated through N_2_ adsorption–desorption isotherms. The surface areas and the PSD results were determined by the BET and BJH methods, respectively. Both m-C and WC/m-C supports exhibited Type IV isotherms (Figure S3a,b), indicating the presence of mesopores. The BET surface areas are 1354 m^2^g^−1^ for m-C and 422 m^2^g^−1^ for WC/m-C, significantly higher than the surface area of the widely used carbon black support (Vulcan XC-72R, 250 m^2^g^−1^). The PSD results (Figure S3c,d) show that the average pore sizes of m-C and WC/m-C are 14.8 nm and 13.5 nm, respectively, aligning with the size of the silica template and highlighting the effective role of Ludox HS-40 as a pore-directing agent. These characterization results indicate that our synthetic method is suitable for fabricating mesoporous catalyst supports (m-C and WC/m-C) with high surface areas, facilitating easier metal loading and, consequently, use as PEMFC electrocatalysts.

The electrochemical properties of the prepared m-C and WC/m-C were investigated by cyclic voltammetry (CV) in an N_2_-purged 1M H_2_SO_4_ solution (Figure S4). In the CV curve of m-C, no hydrogen desorption region (HDR) or hydrogen adsorption region (HAR) is observed in the 0–0.4 V range. However, WC/m-C shows small but distinct currents at the HDR and HAR, indicating that WC possesses hydrogen oxidation activity.

### 3.2. Physical and Electrochemical Properties of Pd Nanoparticles Loaded on the Supports

Pd nanoparticles were deposited onto the m-C (Pd/m-C), and the WC/m-C (Pd/WC/m-C) supports as HOR catalysts. For comparison, typical carbon black (Vulcan XC-72R) was also used as a support for Pd (Pd/C). The NH_3_-mediated polyol method was used for the deposition of Pd nanoparticles. The conventional polyol process using an ethylene glycol solution possesses various advantages in metal nanoparticle synthesis, such as precise control of size distribution [38]. However, for Pd nanoparticles, additional surfactants are typically required to prevent hydrolysis and the formation of Pd (OH)_x_ precipitates before reduction in the ethylene glycol solution. NH_3_ has a strong complexation ability with Pd, preventing Pd (OH)_x_ precipitation even in the absence of surfactants [25]. Therefore, we adopted the NH_3_-mediated polyol method to load Pd nanoparticles on the supports.

**Figure 1:**
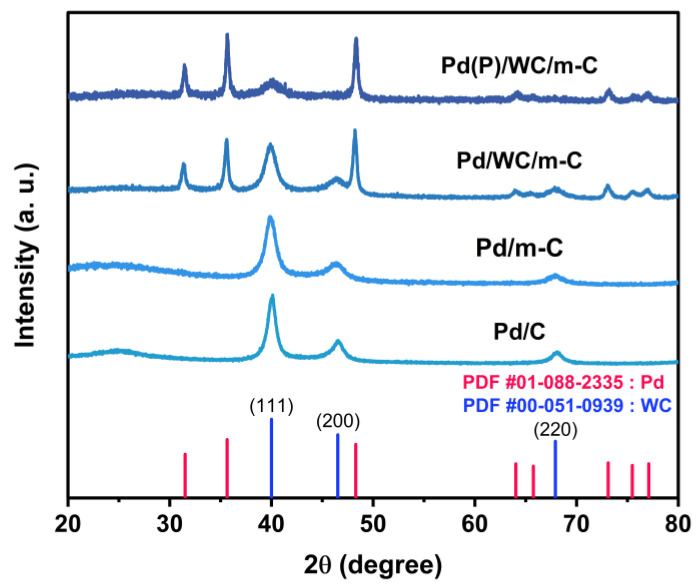
XRD patterns of Pd/C, Pd/m-C, Pd/WC/m-C, and Pd(P)/WC/m-C.

The XRD patterns of the Pd/C, Pd/m-C, and Pd/WC/m-C catalysts are presented in Figure 1. The diffraction peak at ca. 25° corresponds to the (002) crystal face of carbon. Other common diffraction peaks observed at 39.7°, 45.8°, and 67.8° in all samples correspond to the (111), (200), and (220) planes of the face-centered cubic structure of Pd. The characteristic peaks of hexagonal WC are also evident in the Pd/WC/m-C sample. Notably, the Pd peaks on m-C and WC/m-C are broader than those of the Pd/C catalyst, indicative of the smaller average size of the Pd crystallites. Indeed, average crystallite sizes estimated using the Scherrer equation were 13.9 nm for Pd/C, 9.8 nm for Pd/m-C, and 9.6 nm for Pd/WC/m-C.

Figure S5 shows the STEM images of the Pd nanoparticles deposited on the supports. The average Pd particle sizes were consistent with the XRD data in Table 1. Furthermore, less aggregation of Pd nanoparticles was observed when loaded onto m-C compared to carbon black. The higher surface area of m-C likely contributes to the smaller size and improved dispersion of the Pd nanoparticles.

**Figure 2:**
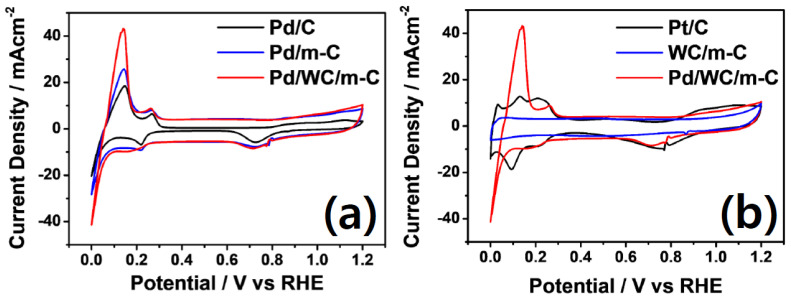
Cyclic voltammograms of (**a**) Pd/WC/m-C vs. Pd/m-C and Pd/C, and (**b**) Pd/WC/m-C vs. Pt/C and WC/m-C.

Because of the smaller size and better dispersion of Pd nanoparticles, the Pd/m-C catalyst demonstrated higher current densities in both the HDR and HAR regions than Pd/C, as shown in Figure 2a. Consequently, the electrochemically active surface area (ECSA) values for Pd/m-C, calculated from HDR and HAR measurements, were higher, indicating enhanced HOR activity. Notably, the Pd/WC/m-C catalyst exhibited even higher current densities in these regions than Pd/m-C. Specifically, ECSA values for Pd/WC/m-C are 99.07 m^2^g^−1^ (by HDR) and 61.37 m^2^g^−1^ (by HAR), surpassing the 65.62 m^2^g^−1^ (by HDR) and 46.47 m^2^g^−1^ (by HAR) for Pd/m-C (Table 2). Despite the larger surface area of the support in Pd/m-C compared to Pd/WC/m-C, the HOR activity of Pd/WC/m-C was approximately 1.5-fold higher than that of Pd/m-C. Furthermore, Pd/WC/m-C showed higher ECSA values than the commercial Pt/C catalysts, as depicted in Figure 2b. This enhancement can be attributed to a strong metal–support interaction and a synergistic effect between Pd and WC, as reported in previous studies [39,40,41,42].

### 3.3. Physical and Electrochemical Properties of Phosphorus-Modified Pd Nanoparticles Loaded on the Supports

Although the HOR activity of Pd/WC/m-C was superior to that of the typical Pt/C (E-TEK) catalyst, there is potential for further improvement, given that the Pd particles in Pd/WC/m-C were still relatively large (~10 nm) compared to those in Pt/C (2–3 nm). Recent methods have been developed to produce smaller Pd nanoparticles by introducing additional metallic or non-metallic elements [40,41,42]. Among them, P-modified Pd demonstrated smaller particle sizes and increased activities in oxygen reduction and formic acid/ethanol oxidation reactions [43].

Anticipating that smaller Pd nanoparticles could enhance hydrogen oxidation activity, we prepared Pd(P) nanoparticles on the support materials using the same NH_3_-mediated polyol reduction method, except that sodium hypophosphite was added to the ethylene glycol solution [26,33]. Figure S6 compares the XRD patterns of Pd(P) and Pd on various supports. The impact of adding a P source on the size of Pd nanoparticles is clearly observed across all supports (Figure S6a–c); the Pd(P) nanoparticles exhibited much broader XRD patterns compared to their unmodified Pd counterparts. Indeed, the estimated Pd(P) particle sizes were significantly smaller than those of Pd, as listed in Table 1. Notably, the size of Pd particles was reduced from 9.6 nm to 3.6 nm after introducing P into the WC/m-C support.

**Figure 3:**
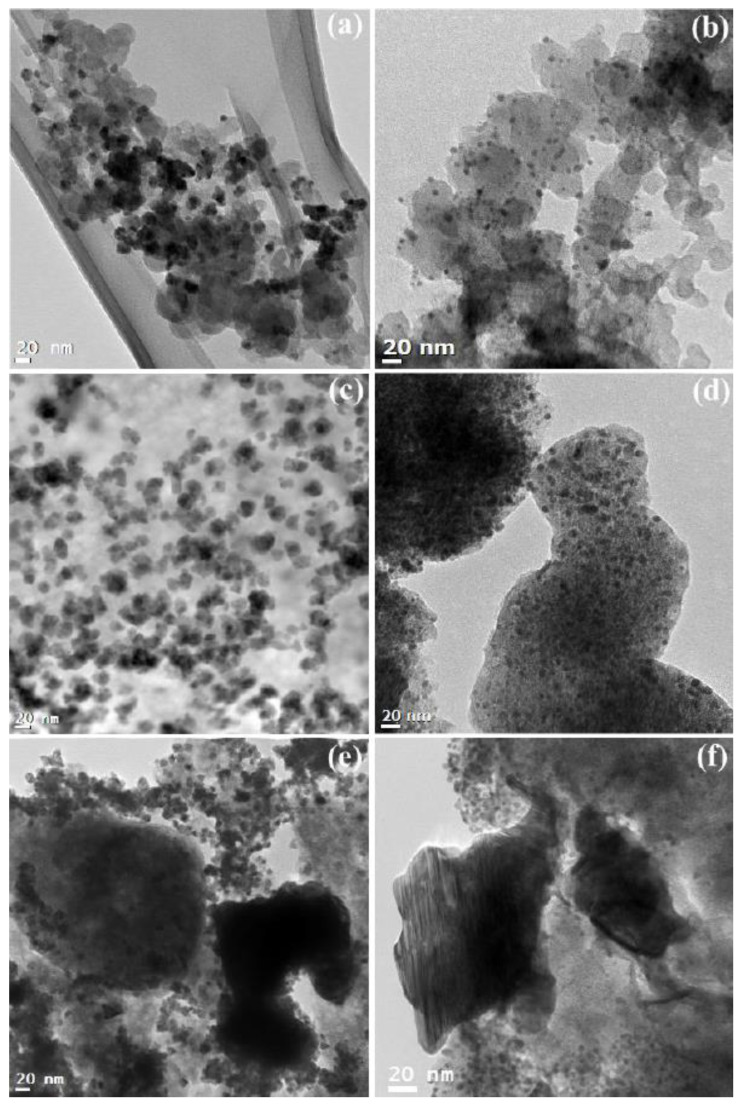
TEM images of (**a**) Pd/C, (**b**) Pd(P)/C, (**c**) Pd/m-C, (**d**) Pd(P)/m-C, (**e**) Pd/WC/m-C, and (**f**) Pd(P)/WC/m-C.

Figure 3 presents TEM images of the Pd(P) nanoparticles on the supports. These images confirm that the Pd(P) nanoparticles are significantly smaller than the unmodified Pd nanoparticles on the same supports. Additionally, the dispersion of Pd particles is enhanced, with reduced aggregation. The sizes of Pd and Pd(P) nanoparticles, calculated based on XRD peaks and TEM images, are listed in Table 1.

**Figure 4:**
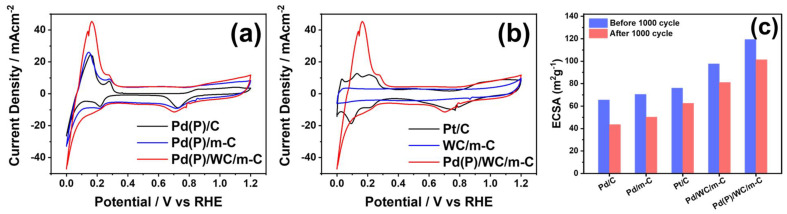
(**a**) Cyclic voltammograms of Pd(P)/C, Pd(P)/m-C, and Pd(P)/WC/m-C. (**b**) Cyclic voltammograms of Pt/C, WC/m-C, and Pd(P)/WC/m-C. (**c**) Comparison of ECSA before and after 1000 CV cycles, measured by CO stripping.

The CV results presented in Figure 4a,b reflect the reduced Pd particle size or increased active area. Compared to unmodified Pd catalysts, the current density of Pd(P) catalysts increased for all supports, as shown in Figure S7a–c. The ECSA of Pd and Pd(P) catalysts, calculated from the hydrogen desorption region (HDR) and hydrogen adsorption region (HAR), are listed in Table 2. An apparent deviation from the general trend is that the calculated ECSA values for Pd(P)/m-C were smaller than those for Pd(P)/C, although Pd(P)/m-C exhibited higher current density than Pd(P)/C. The double-layer capacitance of m-C is larger than that of carbon black due to its higher surface area, which causes the baselines of the cyclic voltammograms to shift up and down, thereby reducing the areas of the hydrogen adsorption and desorption peaks. Furthermore, Pd(P)/WC/m-C shows the best HOR activity, similar to Pd/WC/m-C (Figure 2), with the activity derived from the more common hydrogen desorption peak being more than twice as high as that of the commercial Pt/C (E-TEK).

To further confirm the enhanced ECSA value of Pd(P)/WC/m-C, CO stripping voltammetry measurements were conducted. A previous report pointed out that it is challenging to accurately measure the ECSA of Pd using hydrogen adsorption/desorption peaks alone due to the several limitations of H_upd_ (including H adsorption/intercalation and desorption/deintercalation in Pd) [44]. CO adsorption occurs specifically on the active sites of Pd, making it a more direct and surface-specific method for assessing ECSA. Accordingly, the CO stripping peaks can provide more reliable information [45]. The Pd(P)/WC/m-C recorded an ECSA value of 119.34 m^2^g^−1^, outperforming the other catalysts (Figure S8), aligning with Table S1.

To investigate durability of the catalysts, ECSA (by CO stripping) values were compared before and after 1000 cycles of CV (0–1.0 V). After 1000 cycles, Pd(P)/WC/m-C demonstrated better durability than Pd/WC/m-C, retaining a higher ECSA value of 101.38 m^2^g^−1^, compared to 81.18 m^2^g^−1^ (81.7% retention) of Pd/WC/m-C (Figure 4c and Figure S8).

To investigate why P-based modification results in smaller Pd particle sizes, we conducted XPS, ICP, and TEM-EDX analyses. The significantly higher phosphorus concentrations measured from surface-sensitive XPS compared to ICP, as shown in Table S2, indicate that P predominantly exists on the surface rather than in the interior of the Pd(P) structure. The TEM-EDX elemental mapping of Pd(P)/WC/m-C, presented in Figure S9, shows that P is distributed across all the participating elements of C, W, and Pd. Additionally, there is no evidence of P incorporation into the Pd lattice in the XRD patterns of Pd(P) catalysts displayed in Figure S6. The binding energy of the P 2p XPS peak, shown in Figure S10, was 133.2 eV, indicating that most of the phosphorus exists as a phosphate (PO_4_^3−^) group on the surface of WC/m-C [46,47,48,49].

What, then, is the role of the phosphate group in forming such small Pd nanoparticles? It is instructive to first consider the behavior of Pt dispersion on carbon black supports, which has been extensively studied. Rodriguez-Reinoso summarized the interactions between the carbon surface and metal ions or anions [50]. Maximum catalyst dispersion and resistance to sintering can arise from several factors, but most relevant to our case is the interaction between the precursor ionic species and the carbon surface. Oxidation of the carbon typically renders the surface more acidic and, consequently, negatively charged over a wide pH range. This results in the electrostatic repulsion of PtCl_6_^2−^ anions and the favoring of electrostatic attraction towards [Pt (NH_3_)_4_]^2+^ cations, thereby enhancing metal dispersion. In our study, phosphate groups (PO_4_^3−^) are present on the surface of the support materials. During the Pd(P) loading, NH_4_OH solution was added to make a Pd-NH_3_ complex and to avoid Pd (OH)_x_ precipitation before reduction in ethylene glycol. Thus, the cationic [Pd (NH_3_)_4_]^2+^ is formed, and the surface state remains negative due to the phosphate groups and the basic reducing conditions provided by NaOH. In this case, the attractive interaction between the Pd precursor and the negatively charged surface promotes maximal dispersion of Pd on the support. Consequently, the addition of sodium hypophosphite during the Pd loading process via the NH_3_-mediated polyol reduction method leads to the formation of small, highly dispersed Pd nanoparticles.

## 4. Conclusions

In the present work, we aimed to enhance the electrochemical activity of Pd/WC in the HOR to assess its potential as a Pt-free anode of PEMFC. Initially, we prepared WC-mesoporous carbon composite materials as supports to leverage the high surface area of carbon. Subsequently, we deposited Pd nanoparticles onto the composite using an NH_3_-mediated polyol reduction method. The resulting Pd/WC/m-C catalyst exhibited higher HOR activity comparable to that of commercial Pt/C (E-TEK). This result can be attributed to strong metal–support interactions and a synergistic effect between Pd and WC. Given that the Pd particles in this catalyst were relatively large (ca. 10 nm), we synthesized Pd(P) nanoparticles by adding sodium hypophosphite during the same polyol reduction process. This modification yielded Pd(P) particles ca. 4 nm in size, and the resultant Pd(P)/WC/m-C catalyst demonstrated remarkable HOR activity, approximately twice as high as that of the commercial Pt/C catalyst (E-TEK). The electrostatic interaction between the Pd precursor, [Pd(NH_3_)_4_]^2+^, and the surface group, PO_4_^3−^, facilitated the formation of smaller Pd nanoparticles and maximized Pd dispersion on the supports. This result underscores the potential of non-platinum catalysts to achieve high performance in HOR applications, demonstrating greater activity than commercial catalysts and indicating their viability as replacements for Pt/C catalysts as anodes in PEMFCs.

## Figures and Tables

**Table 1:** Average particle sizes of Pd and Pd(P) catalysts from the Gaussian fitted XRD peaks of Pd (200) using the Scherrer equation and TEM images.

	XRD [nm]	TEM [nm]		XRD [nm]	TEM [nm]
Pd/C	13.9	14.5	Pd(P)/C	5.7	6.3
Pd/m-C	9.8	10.1	Pd(P)/m-C	4.4	5.1
Pd/WC/m-C	9.6	9.8	Pd(P)/WC/m-C	3.6	4.2

d = kλ/β × cosθ (k = 0.9, λ = 1.5406 Å).

**Table 2:** Electrochemical activity of electrocatalysts.

	ECSA [m^2^g^−1^] *			ECSA [m^2^g^−1^]	
	HDR	HAR		HDR	HAR
Pt/C (ETEK)	68.93	74.00			
Pd/C	65.16	43.05	Pd(P)/C	92.67	63.78
Pd/m-C	65.62	46.47	Pd(P)/m-C	78.50	60.87
Pd/WC/m-C	99.07	61.37	Pd(P)/WC/m-C	155.44	96.82

* ECSA was calculated by Q_H_/[Pt] × C_ML_, where Q_H_ = the charge exchanged during the electro-adsorption (HAR) or desorption (HDR) of hydrogen on Pt (mCcm^−2^), [Pt] = the Pt loading (mgcm^−2^), and C_ML_ = the charge needed for the oxidation of a single layer of hydrogen on a smooth Pt surface (0.21 mCcm^−2^).

## Data Availability

Data are contained within the article or Supplementary Materials.

## Supplementary Materials

The following supporting information can be downloaded at: https://www.mdpi.com/article/10.3390/nano14121024/s1. Figure S1: XRD patterns of m-C and WC/m-C; Figure S2: TEM images of (a,b) m-C and (c,d) WC/m-C; Figure S3: N_2_-sorption isotherms for (a) m-C and (b) WC/m-C. Pore size distribution graphs for (c) m-C and (d) WC/m-C; Figure S4: Cyclic voltammograms of m-C and WC/m-C in 1M H_2_SO_4_; Figure S5: STEM images of (a,b) Pd/C, (c) Pd/m-C and (d) Pd/m-C; Figure S6: XRD patterns of (a) Pd/C vs. Pd(P)/C, (b) Pd/m-C vs. Pd(P)/m-C, (c) Pd/WC/m-C vs. Pd(P)/WC/m-C, and (d) Pd(P) on various supports; Figure S7: Cyclic voltammograms of (a) Pd/C vs. Pd(P)/C, (b) Pd/m-C vs. Pd(P)/m-C, (c) Pd/WC/m-C vs. Pd(P)/WC/m-C, Pd/WC/m-C vs. Pd(P)/WC/m-C after 1000 potential cycles; Figure S8: CO stripping cyclic voltammograms (CVs) of the sample; Figure S9: TEM-EDX mapping images for (a) TEM image of Pd(P)/WC/m-C, (b) Pd element, (c) W element, (d) C element and (e) P element; Figure S10: XPS P 2p spectrum of Pd(P)/WC/m-C.; Table S1: The electrochemical surface area (ECSA) value of samples before and after CV 1000cycles; Table S2: Phosphorus concentration (atomic %) of XPS and ICP.
