# Supplementary material for: Phosphorus-Modified Palladium and Tungsten Carbide/Mesoporous Carbon Composite for Hydrogen Oxidation Reaction of Proton Exchange Membrane Fuel Cells
Source: Nanomaterials (Basel). 2024 Jun 13;14(12):1024. doi: 10.3390/nano14121024 (PMC11206704; doi:10.3390/nano14121024)
Supplement: Supplementary file 1 [file nanomaterials-14-01024-s001.zip › nanomaterials-3035524-supplementary.pdf]

Supplementary Materials

Phosphorus-modified palladium and tungsten carbide/mesoporous carbon composite for hydrogen oxidation reaction of proton exchange membrane fuel cells

Ganghong Bae[1,†], Woo Jin Byun[2,†] , Jin Ho Lee[2,†], Min Hee Lee[2], Yeji Choi[3], Jae Young Kim[4,*] and Duck Hyun Youn[3,*]

[1]Department of Chemical Engineering, Pohang University of Science & Technology (POSTECH), Pohang 37673, South Korea

[2]School of Energy and Chemical Engineering, Ulsan National Institute of Science and Technology (UNIST), Ulsan 44919, South Korea

3Department of Chemical Engineering, Department of Integrative Engineering for Hydrogen Safety, Kangwon National University, Chuncheon 24341, South Korea

[4]Korea Research Institute of Chemical Technology, Daejeon 34114, South Korea

[†] These authors contributed equally to this work

*Correspondence: jaeykim@krict.re.kr (J.Y.K.); youndh@kangwon.ac.kr (D.H.Y.)

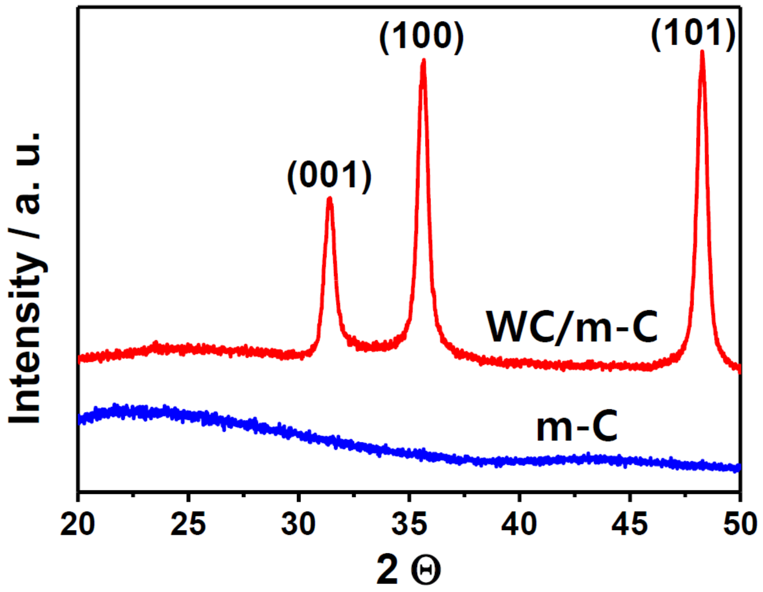

Figure S1. XRD patterns of m-C and WC/m-C.

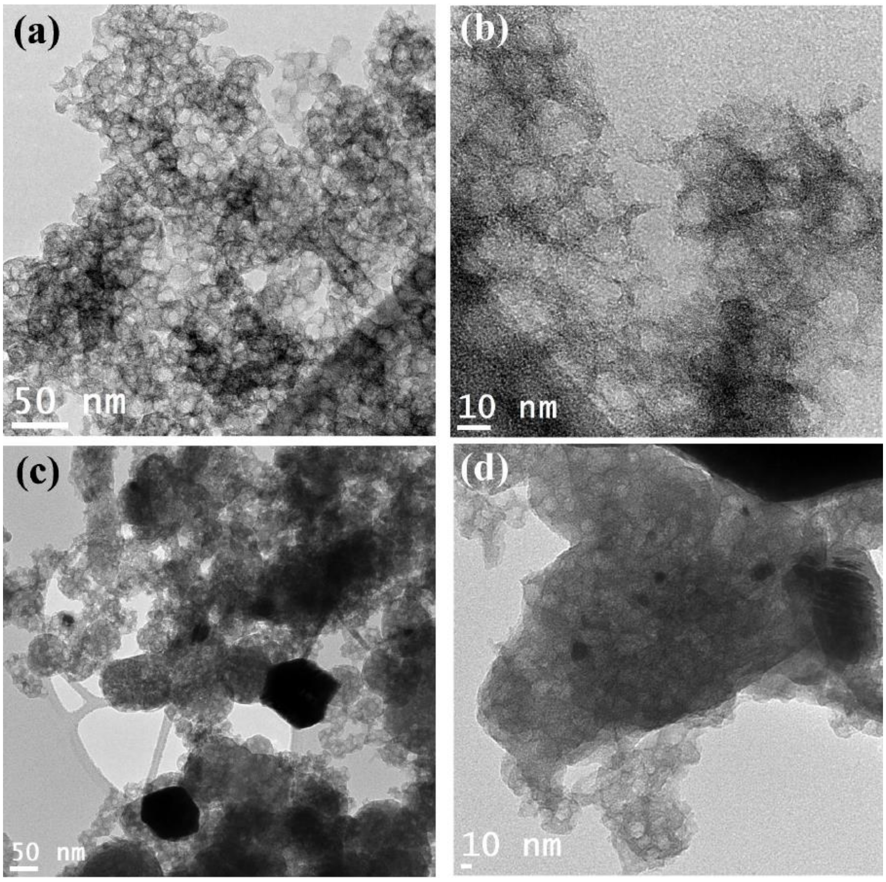

Figure S2. TEM images of a), b) m-C, and c), d) WC/m-C.

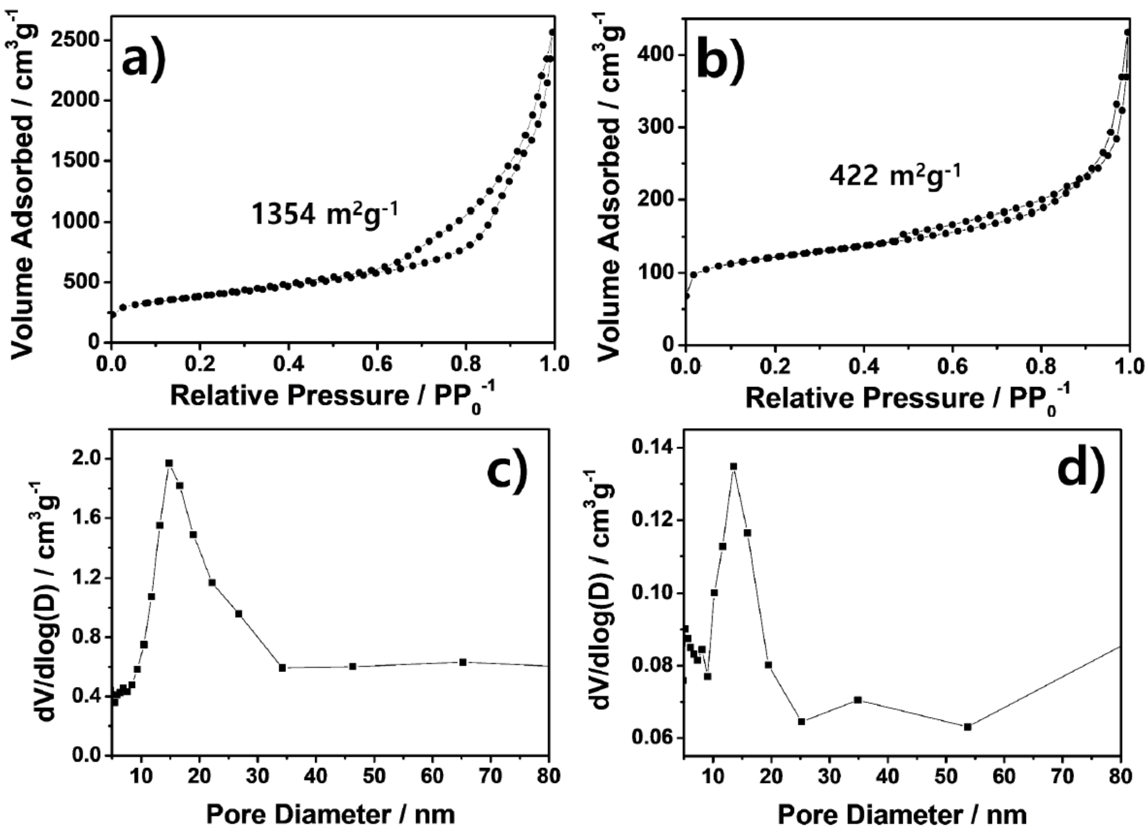

Figure S3. N_2-sorption isotherms for a) m-C and b) WC/m-C. Pore size distribution graphs for c) m-C and d) WC/m-C.

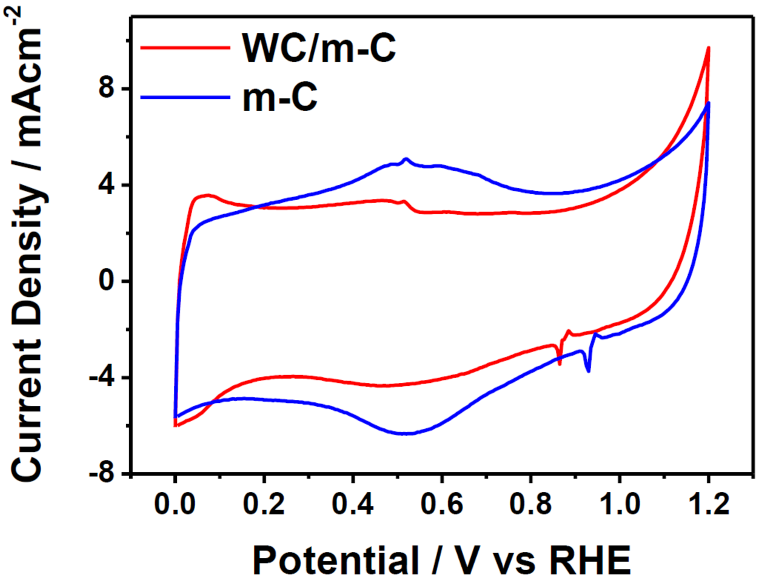

Figure S4. Cyclic voltammograms of m-C and WC/m-C in 1M H_2SO_4.

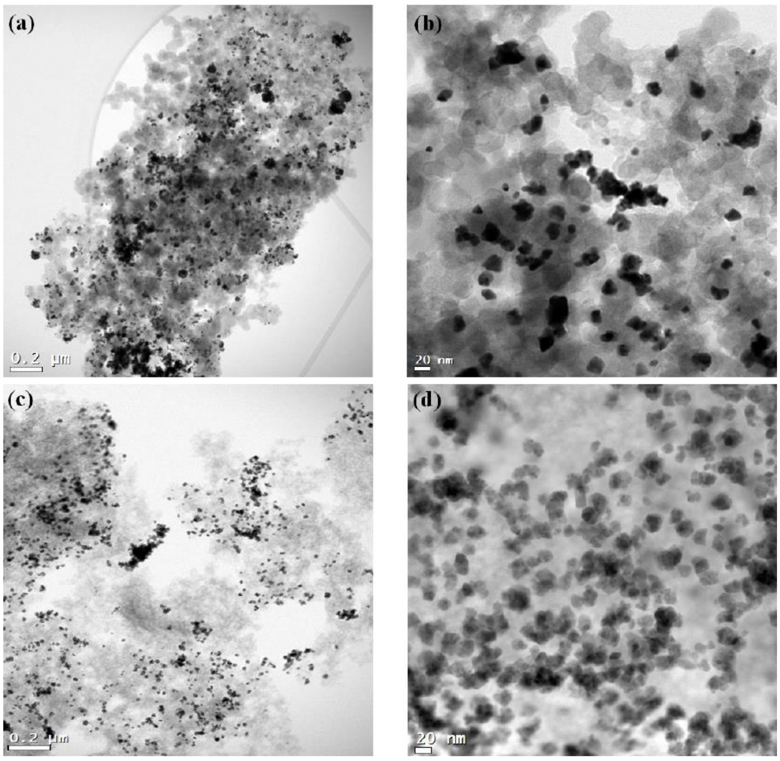

Figure S5. STEM images of a, b) Pd/C, c) Pd/m-C and d) Pd/m-C.

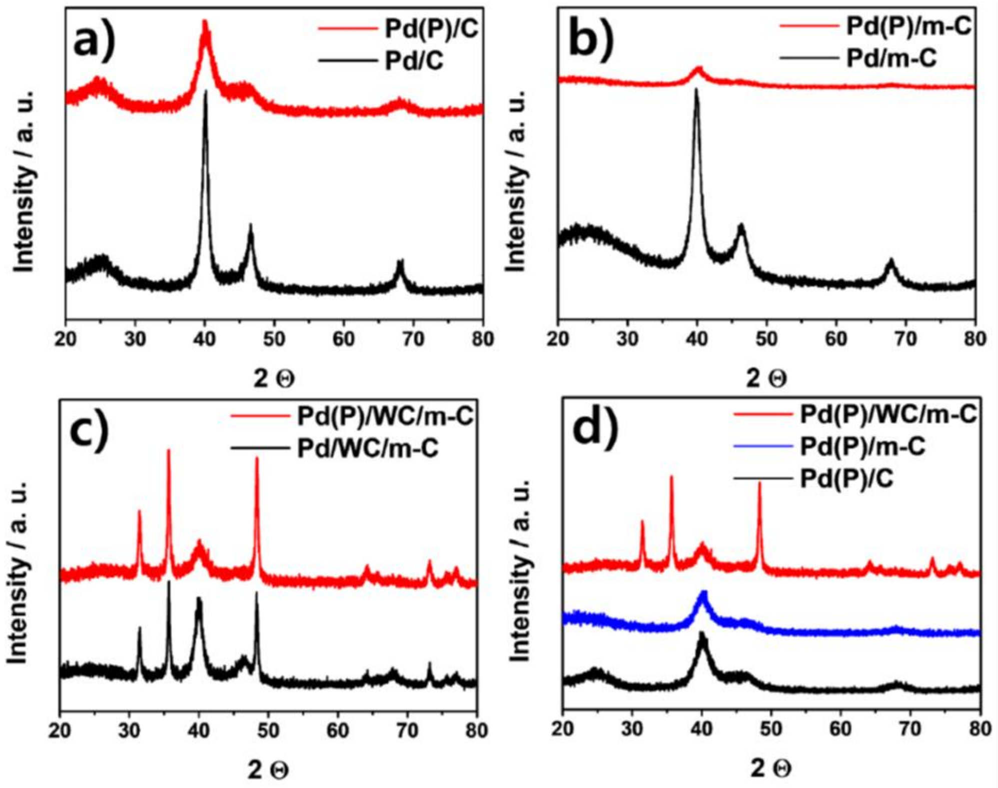

Figure S6. XRD patterns of a) Pd/C vs Pd(P)/C, b) Pd/m-C vs Pd(P)/m-C, c) Pd/WC/m-C vs Pd(P)/WC/m-C, and d) Pd(P) on various supports.

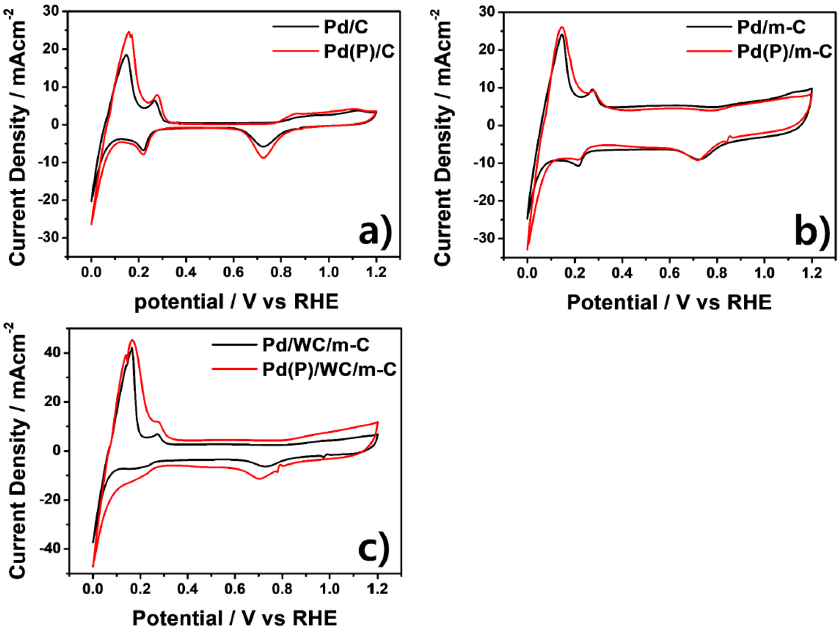

Figure S7. Cyclic voltammograms of a) Pd/C vs Pd(P)/C, b) Pd/m-C vs Pd(P)/m-C, and c) Pd/WC/m-C vs Pd(P)/WC/m-C.

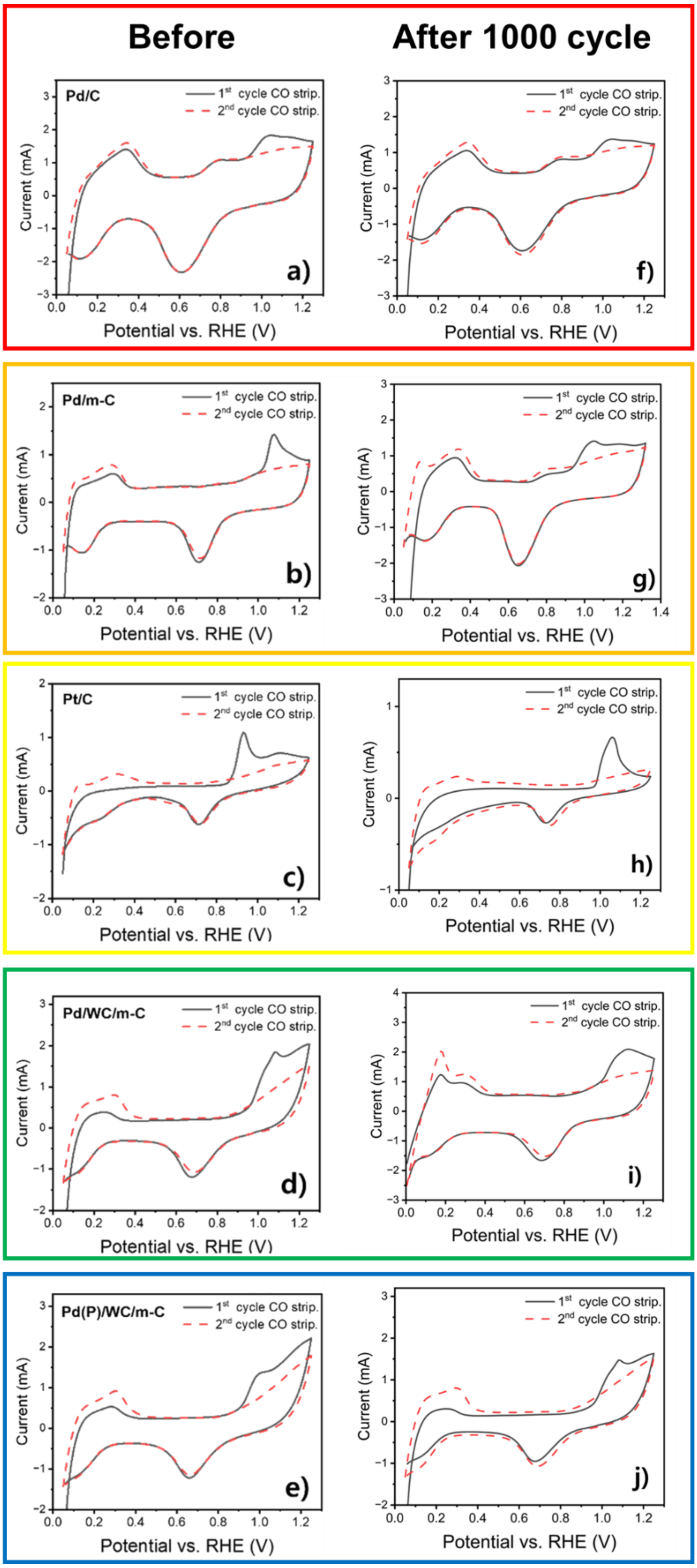

Figure S8: CO stripping cyclic voltammograms (CVs) before (left) and after (right) 1000 cycles. (a, f) Pd/C, (b, g) Pd/m-C, (c, h) Pt/C, (d, i) Pd/WC/m-C, and (e, j) Pd(P)/WC/m-C.

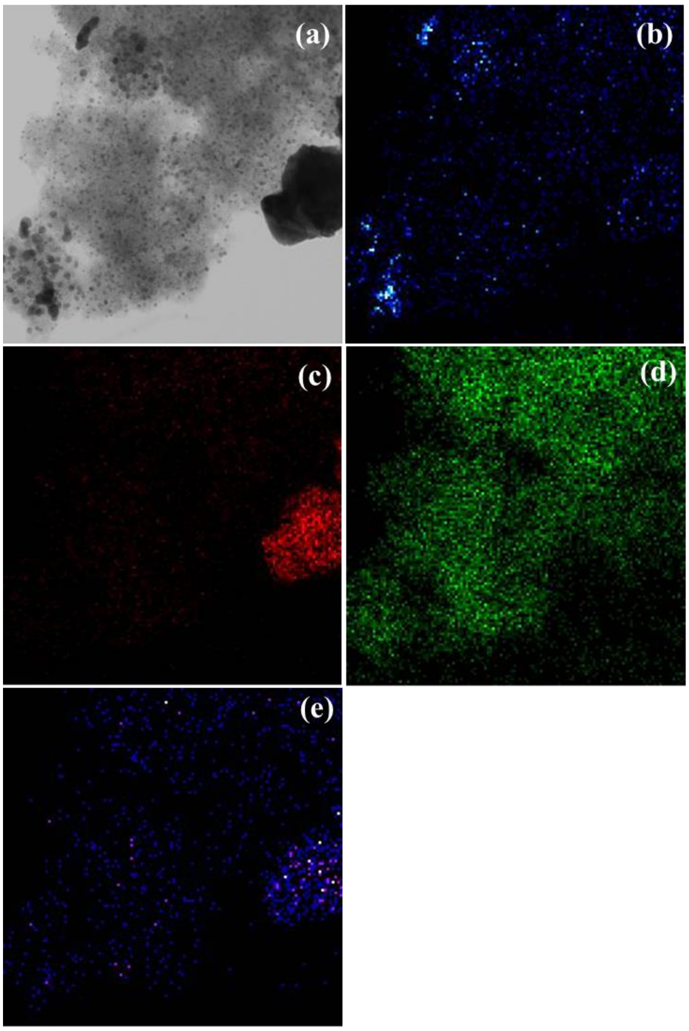

Figure S9. TEM-EDX mapping images for (a) TEM image of Pd(P)/WC/m-C, (b) Pd element, (c) W element, (d) C element and (e) P element.

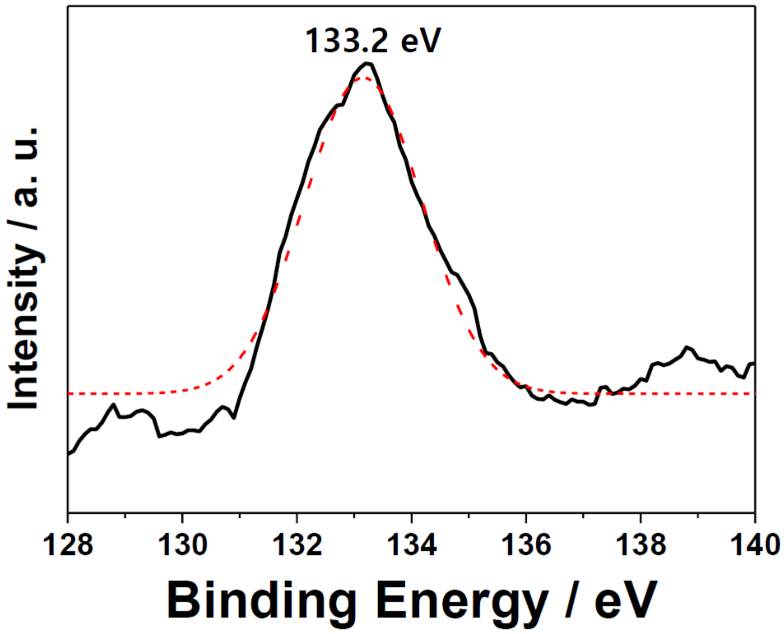

Figure S10. XPS P 2p spectrum of Pd(P)/WC/m-C.

Table S1. The electrochemical surface area (ECSA) value of samples before and after CV 1000cycles.

Catalyst	Pd/C	Pd/m-C	Pt/C	Pd/WC/ m-C	Pd(P)/WC /m-C
$ECSA_{before}$ (m^2/g^{-1})	65.43	70.44	76.96	97.66	119.35
$ECSA_{after}$ (m^2/g^{-1})	43.58	50.26	62.49	81.18	101.48
Retention (%)	54.87	63.85	80.59	81.73	84.05

Table S2. Phosphorus concentration (atomic %) of XPS and ICP.

.	Total P [at %]	
	XPS	ICP[a)]
Pd(P)/C	0.8	0.04
Pd(P)/m-C	0.6	0.04
Pd(P)/WC/m-C	1.5	0.28

[a)]ICP data are calculated from wt %.
